# Topology analysis and visualization of *Potyvirus* protein-protein interaction network

**DOI:** 10.1186/s12918-014-0129-8

**Published:** 2014-11-20

**Authors:** Gabriel Bosque, Abel Folch-Fortuny, Jesús Picó, Alberto Ferrer, Santiago F Elena

**Affiliations:** Institut Universitari d’Automàtica i Informàtica Industrial, Universitat Politècnica de València, Camí de Vera s/n, 46022 València, Spain; Departamento de Estadística e Investigación Operativa Aplicadas y Calidad, Universitat Politècnica de València, Camí de Vera, s/n, Edificio 7A, 46022 València, Spain; Instituto de Biología Molecular y Celular de Plantas, Consejo Superior de Investigaciones Científicas-Universitat Politècnica de València, Campus UPV CPI 8E, Ingeniero Fausto Elio s/n, 46022 València, Spain; The Santa Fe Institute, Santa Fe, NM 87501 USA

**Keywords:** Amplification of perturbations, Network biology, *Potyvirus*, Protein interaction network, Systems biology, Virology

## Abstract

**Background:**

One of the central interests of Virology is the identification of host factors that contribute to virus infection. Despite tremendous efforts, the list of factors identified remains limited. With omics techniques, the focus has changed from identifying and thoroughly characterizing individual host factors to the simultaneous analysis of thousands of interactions, framing them on the context of protein-protein interaction networks and of transcriptional regulatory networks. This new perspective is allowing the identification of direct and indirect viral targets. Such information is available for several members of the Potyviridae family, one of the largest and more important families of plant viruses.

**Results:**

After collecting information on virus protein-protein interactions from different potyviruses, we have processed it and used it for inferring a protein-protein interaction network. All proteins are connected into a single network component. Some proteins show a high degree and are highly connected while others are much less connected, with the network showing a significant degree of dissortativeness. We have attempted to integrate this virus protein-protein interaction network into the largest protein-protein interaction network of *Arabidopsis thaliana*, a susceptible laboratory host. To make the interpretation of data and results easier, we have developed a new approach for visualizing and analyzing the dynamic spread on the host network of the local perturbations induced by viral proteins. We found that local perturbations can reach the entire host protein-protein interaction network, although the efficiency of this spread depends on the particular viral proteins. By comparing the spread dynamics among viral proteins, we found that some proteins spread their effects fast and efficiently by attacking hubs in the host network while other proteins exert more local effects.

**Conclusions:**

Our findings confirm that potyvirus protein-protein interaction networks are highly connected, with some proteins playing the role of hubs. Several topological parameters depend linearly on the protein degree. Some viral proteins focus their effect in only host hubs while others diversify its effect among several proteins at the first step. Future new data will help to refine our model and to improve our predictions.

**Electronic supplementary material:**

The online version of this article (doi:10.1186/s12918-014-0129-8) contains supplementary material, which is available to authorized users.

## Background

*Potyvirus* is the mayor genus in the Potyviridae family, accounting for 30% of all known plant viruses, with more than 180 members. Many potyviruses are important pathogens of agricultural crops. They are able to infect a wide range of mono- and dicotyledonous plant species [1], causing symptoms that severely reduce the yield and quality of crops. The economic impact of these viruses on agriculture is well-documented [2]. Some examples of potyviruses are *Plum pox virus* (PPV), *Soybean mosaic virus* (SMV), *Turnip mosaic virus* (TuMV), and *Tobacco etch virus* (TEV) [3].

Potyvirus virions are flexuous and rod-shaped, 680 to 900 nm long and 11 to 15 nm wide [4]. Potyviruses have a single-stranded, positive-sense RNA genome of approximately 10 kilobases (kb). They contain two open reading frameworks (ORF). The first one is a long ORF which is translated into a large polyprotein, which subsequently self-processes into 10 mature functional proteins: P1, a serine protease also involved in enhancement of polyprotein translation; HC-Pro, a protease with RNA silencing suppressor activity that also mediates aphid transmission; P3, which play a role in cell-to-cell movement; 6 K1, a small peptide that links the replication complexes to ER membranes; CI, an RNA helicase with ATPase activity; 6 K2, another small peptide of unknown function; VPg, linked to the 5′ end of the genome; NIaPro, the mayor protease; NIb, the RNA-dependent RNA polymerase; and CP, the capsid protein [5]. The second ORF is a small one embedded within the P3 coding region and results from +2 frame-shift [6,7]. This recently discovered ORF encodes the eleventh protein, P3N-PIPO, also involved in cell-to-cell movement. Much research in the last two decades has focused on understanding the functions of the different potyvirus proteins during the virus life cycle. Rapid rise of academic interest in this topic followed the complete sequencing of the first two potyviruses: TEV [8] and *Tobacco vein mottling virus* (TVMV) [9]. Many excellent reviews have been published since then [4,10]; some addressing particular issues such as protein function [11], polyprotein processing [12,13], cellular localization [14] and genome structure [1].

During the last decade there has been an increasing number of studies of protein-protein interactions (PPIs) and the effect that these interactions cause on a wide range of biological processes [15]. PPIs are defined as physical contacts that take place in cells through molecular docking [16]. Proteins work typically linked to other molecules including lipids, nucleic acids or other proteins [17]. Biological activity usually arises from the association of several proteins, which form protein complexes. In viruses, interactions between proteins play vital roles in many processes during infection such as virus trafficking between the nucleus and the cytoplasm, formation of replication complexes, assembly of virions, or virus transmission to other cells. Traditionally, PPIs have been studied using methods such as coimmunoprecipitation or chromatography [18]. However, over the past decade two experimental strategies have been used to detect these interactions: yeast two-hybrid (Y2H) [17,19,20] and affinity purification coupled with mass spectrometry (AP-MS) [21]. Additionally, bimolecular fluorescence complementation (BiFC) [22,23] has grown in popularity during the last few years because it allows PPI visualization in living cells, which is a key aspect to understand their cellular functions.

PPIs form networks of linked proteins which are called consequently protein-protein interaction networks (PPINs) [16]. PPINs can be seen as a visual representation of the complete map of interactions that a system (pathway, cell, living organism) establishes in a particular moment and for a certain time window. Detection methods (specially Y2H) opened the possibility to tackle protein-protein interactions on a genome wide scale, producing complete PPINs, which have been called interactomes [24-27]. Viral PPINs have also been developed [28,29], revealing quite useful biological information.

The analysis of viral PPINs presents interactions between two proteins of the virus (VVPIs) or interactions between viral proteins and host proteins (VHPIs). These PPINs illustrate a fundamental property of viral proteins: their multifunctionality. Viral proteins usually perform different functions at different stages of the infection cycle. Moreover, their role changes along with the infection process. Thus, detecting VHPIs provides valuable insight into viral mechanisms and processes. VHPIs are responsible of channeling the effect of the virus into the plant. In addition, interactions between host proteins (HHPIs) are also fundamental in order to understand the interplay between virus and host, and the biological consequences once the virus effect starts to propagate across the host PPIN [30].

PPINs, as any other network, may be described and studied from a complex systems point of view. Over the past fifteen years many researchers have focused on developing tools and frameworks to study, categorize and understand networks [31-34]. Some work has been done applying network theory to biological networks, developing a new discipline or approach called Network Biology [35-38]. An excellent and updated review on topology of interaction networks may be found in [39]. Some studies have dealt with the topological properties and features of PPINs [33,40-42], however just a few have focused on viral PPINs [5,29,43]. Viral infection is a complex process and it requires a systems approach to be fully described. A more detailed and systematic understanding of how viral proteins interact with each other, and with host proteins, might allow developing new drugs and treatments that block the viral replication in a more efficient and durable manner. Unfortunately, there remains a need for a much deeper understanding of viral PPINs using the topological tools and methods developed by complex systems and network science.

Following this major current approach, in this study we present a topological analysis of the potyvirus PPIN constructed by integrating data from several different species of potyvirus. We also study the VHPIs using the complete *Arabidopsis thaliana* PPIN. Furthermore we describe and quantify the effect that the viral network and each of its components has on the host interactome. Finally, we propose new ways to visually represent the VHPI network (VHPIN).

## Methods

### Data collecting

All currently available potyvirus VVPI datasets were gathered as a first step. These data were obtained from six different articles published over the last decade [44-49]. This initial dataset, shown in Additional file 1, is the starting point of the subsequent analysis. An overview of the data is shown in Table 1. 681 PPIs were tested and 194 PPIs were detected among the 11 viral proteins from eight different viruses: *Plum pox virus* (PPV), *Soybean mosaic virus* (*Pinellia ternate* isolate, SMV-P), *Shallot yellow strip virus* (onion isolate, SYSV-O), *Potato virus A* (PVA), *Pea seed-borne mosaic virus* (PSbMV), *Soybean mosaic virus* (G7H strain, SMV-G7H) and *Clover yellow vein virus* (CIYVV). Some of the Y2H original studies included information about the relative intensity of each interaction, represented by a higher or lower number of colonies appearing after an incubation time. However, integrating the intensity data is not straightforward because it depends on some experimental variables such as sampling schemes, growth variables or environment conditions. Furthermore, differences in normalization methods, categorization and batch effects also contribute to make comparisons difficult. Especially problematic was the inclusion of the P3N-PIPO protein. This protein was discovered and characterized only recently and, therefore, it was not included in some of the studies in which we grounded our work. However, the statistical standardization of the data allows an appropriate representation of P3N-PIPO interactions (see Results and Discussion section, Interaction relevance subsection).

**Table 1:** ***Potyvirus*** **interactions initial dataset**

**Reference**	**Virus**	**Interactions**		**Method**
		**Tested**	**Detected**	
[44]	PPV	105	54	BiFC
[45]	SMV-P	100	39	Y2H
	SYSV-O	100	45	Y2H
[46]	PVA	80	16	Y2H
	PSbMV	56	10	Y2H
[47]	PRSV-P	100	16	Y2H
[48]	SMV-G7H	100	9	Y2H
[49]	CIYVV	40	5	Y2H

It contains data from six different studies and eight different viruses.

The second basic source of data was the *A. thaliana* interactome formed by 12654 interactions and 5127 proteins published in [50] plus the most recently discovered HHPIs (Additional file 2). Although some studies have analyzed the changes produced by virus infection in natural hosts, *A. thaliana* is the standard model host used with viruses belonging to different taxonomic families [5]. The final data source was the group of VHPIs detected between proteins from potyviruses and *A. thaliana* published originally in [5] and later updated (Additional file 3). Therefore the data covers all possible protein interactions: virus-virus (VVPI), virus-host (VHPI) and host-host (HHPI).

### Data integration: interactions, matrices and networks

Integrating data from different sources in a common framework required of statistical standardization and preprocessing. First, each interaction tested in the original studies was collected. Some of them were able to test more interactions than others. In some studies it was not possible to produce enough quantity of a certain protein to test its interactions with the others. In other cases proteins had not been yet discovered when the studies took place so they are obviously absent. Additionally, not all interactions tests resulted in a positive interaction being detected. All detected interactions were collected as well. Tested and detected interactions across all sources were grouped in two common pools (Additional file 4).

The molecular methods used to detect the interactions have an inherent directionality. Experimentally, it is common to swap the fused tags among the pair of proteins to avoid possible structural problems that may interfere with the detecting methods (*e.g.*, Y2H and BiFC). Original studies tested all interactions in two directions, for instance P1 ~ HC-Pro and HC-Pro ~ P1. This produces a problem when only one direction was detected. Since the PPI itself has no directionality (it is a molecular docking phenomenon between two molecules) the disagreement comes from the molecular methods used. Some combinations of fused and viral proteins may be less stable or may block the docking of other proteins. To overcome this, it was assumed that an interaction was valid if it was detected in any of the two directions or in both. This produces symmetry in complementary interactions (P1 ~ HC-Pro and HC-Pro ~ P1) representing the real process of interacting in a clearer and more truthful way.

The next step was to determine which interactions were relevant and which ones were fair representations of the *Potyvirus* genus topology. Given the variability among studies (*e.g.*, virus species and experimental conditions) it is not surprising that some interactions were detected only in one or few studies, while other were pervasive across the entire dataset. On the other hand, the relative scarcity of the data (only 194 interactions detected) made difficult and somewhat useless a more detailed statistical analysis. Even a confidence interval for each interaction with only eight independent values (corresponding to the eight viruses) is not reliable enough. Therefore, a relevance coefficient (*RC*) between the numbers of detected and tested interactions for each pair of proteins was defined. It is reasonable to assume that *RC* is a measure of biological importance. In other words, the more times an interaction has been detected, the higher the probability that this particular interaction is important for the virus to complete its infectious/replication cycle. However, considering the particularities of each method, we weighted percentages for Y2H and BiFC. The latter is closer and much more biologically coherent to natural conditions where potyvirus interactions take place. Therefore, we decided to overweight the only study in which this method was used [44]. Thus, *RC* takes the form *RC* = 100 × (2[*BiFC*] + [*Y2H*])/(*T* +1), where *T* is the number of times that a particular interaction was tested (from 0 to 8), [*BiFC*] is the number of times that a given interaction was detected using the BiFC method (from 0 to 1 because only one study used BiFC) and [*Y2H*] corresponds to the number of times that an interaction was detected using the Y2H methodology (from 0 to 7). The factor of 2 multiplying the [*BiFC*] term is a simple way to overweight this method against the Y2H. Doubling its importance was a compromise solution between being truthful to the particularities of each method and still gathering all the relevant information. *RC* can range then from 0% (the interaction was not detected in any of the studies) to 100% (was detected in every single study). We decided to establish the *RC* threshold for each interaction at the minim value where all nodes were part of a single connected network, which occurred at *RC* = 44%. This choice has biological meaning because is based on the fact that all Potyvirus genomes encode for the eleven proteins and that all these proteins have been reported to interact at least once with each other. Therefore, it is only possible to study this particular system assuming only one connected network, which appears at *RC* = 44%. We decided to set the threshold at this value to include all information considered relevant from our approach. This threshold is data-dependent and therefore can change from network to network. Even with the same dataset it may be changed to satisfy a particular research objective. For instance, setting a higher *RC* makes the analysis focus on the most frequent interactions, which may be interesting in a specific situation. However, lower *RC* than 44% results in a disconnected network with various components. Using the relevant interactions we constructed an interaction matrix with the eleven viral proteins as rows and columns, and the *RC* values for the interactions in each position. Finally, we displayed this matrix visually in a PPIN.

### Network topology

After integrating the data, an exhaustive topological analysis was carried out. First, the protein connectivity aspects of the network were studied: protein degree, *RC* relation with protein degree and assortativity. Then a group of topological parameters (clustering coefficient, closeness centrality, betweenness centrality, and topological coefficient) was calculated for the viral PPIN and its nodes. Finally we carried out an analysis of these topological parameters: their relation with the degree and their cumulative distributions.

The topological analysis of the viral PPIN and its nodes was repeated for those individual virus networks with enough interactions detected to form a complete topology (Table 1): PPV, SMV-P and SYSV-O. All the networks were constructed and the parameters calculated using the software Cytoscape [51] and its network analyzer tool.

### Virus-host interactome

The purpose of the analysis between the virus proteins and the host ones is to achieve an overall better understanding of their relationships and integration, which is pivotal to grasp the infection process. For this, we used an approach to quantify the importance that each viral protein has over the host network. The first order connectivity that each viral protein has with the host proteins can be extracted directly from the data. Starting from each viral protein, and following the host interactome, we calculated how many steps (consecutive interactions) are needed to reach each host protein. At the end, it is possible to map the consecutive steps from the viral protein to the last host protein. This was repeated for all viral proteins and the propagation trajectories produced were plotted.

**Figure 1:**
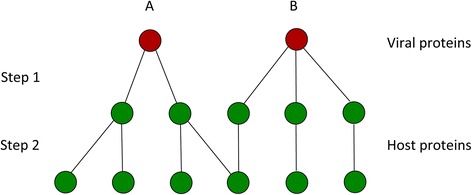
**Examples of steps of interactions.** Step is the measure used to define distance between proteins. In this example **A** would establish 2 interactions in step 1 and 4 in step 2. **B** has 3 in step 1 and 3 in step 2.

Several considerations are here in due, starting from the concept of “distance” in a graph. In this paper we used the simplest distance measure possible, which is the shortest path between two nodes, which comes directly from the adjacency matrix (see Additional file 2) and the cross-interactions or VHPIs (see table in Additional file 3). The minimal measure of distance is called here step. The distance between two proteins interacting directly is one step. The distance between two proteins that interact with another common protein is two steps (Figure 1). From this simple distance we used a metric to qualify the interaction-profile similarity of the viral proteins. Nonetheless, much more complex similarity coefficients [52] can be used as kernels on graphs (*e.g*., exponential diffusion kernel, Laplacian exponential diffusion kernel, or the commute time kernel).

The similarity of the spreading trajectories was compared for every pair of viral proteins with a similarity coefficient or index [53]. The total amount of interactions is 66 (combinations of eleven proteins taken by pairs). We chose the Simpson index (*SI*), which is commonly used in systems biology and network science. It is defined as the proportion of shared nodes relative to the degree of the least connected node: *SI*(*A*,*B*) = |*N*(*A*)∩*N*(*B*)|/*min*(|*N*(*A*)|,|*N*(*B*)|). *SI* changes in each step so the similarity evolves along the whole host interactome. This index offers a quick and insightful way of quantifying the similarity that two viral proteins show in their relationship with the host network.

## Results and discussion

As outlined in the Background section, the aim of this study is to describe and characterize the PPIN of potyviruses using tools and techniques from network science. As mentioned earlier, the study starts from three different datasets: VVPIs, VHPIs and HHPIs. VVPIs allowed us to study the topology of the network, composed exclusively by 11 viral proteins. Next, we evaluated VHPIs and HHPIs and used them to describe and quantify the integration of the viral PPIN within the larger interactome of the host plant.

### VVPI network analysis

In this subsection different aspects of the topology of the network were studied in detail. Y2H and BiFC analysis and Y2H intensity subsections deal with the differences between the detection methods and the nature of the information they provide and the possible consequences for the study. VVPI network construction and visualization subsection shows how the network was visually defined and the last three (Interaction relevance, Protein connectivity and Topological analysis) focus on several aspects of the topological properties of the network.

#### Y2H and BiFC analysis

In this subsection, we compared the results inferred from data generated using the two detection methods. The aim of this comparison was to find out whether a method tends to detect some interactions but not others or, on the contrary, the main interactions were evenly detected by both methods. Interactions detected by both methods will be more reliable than those detected by only one method. The number of observed interactions was classified according to the detection method (Table 1). Some direct remarks can be made just from this simple classification. First, there are 5.4 times more data available from Y2H than from BiFC, which reflects the more recent technological development of BiFC but also introduces a bias towards Y2H-based studies. Despite the lower number of interactions studied using BiFC, the number of positive cases is significantly larger for this technique than for Y2H (Fisher’s exact test *p-value* <0.001), thus proving that BiFC is a more sensitive method. Moreover, BiFC preserves the biological relevance of the interactions detected, since this technique seeks for interactions in plant rather than detect heterologous expression of proteins in yeast cells.

Y2H is an older method, widely used because of its simplicity, speed and its ability to generate interactions at genome level. Y2H also provides a rough measure of interaction intensity given by the number of colonies that grow in each experiment and usually distributed in several ranges (from 1 to 5, from 5 to 10, etc.). Alternatively, BiFC does not provide a quantitative value. Some particularities arise when they are compared. The interaction between CI and P3N-PIPO was only tested and detected by BiFC (due to the recent discovery of the P3N-PIPO protein). Interestingly, the most common interactions are detected by both methods and appear in both networks; out of the 26 most relevant interactions (displayed in Table 2) only three were detected by Y2H but not by BiFC (HC-Pro ~ HC-Pro, HC-Pro ~ NIaPro and HC-Pro ~ VPg). This implies that both methods, although different in scope and sensitivity, offer highly consistent results. This consistency validates our approach of integrating data from both techniques into a single dataset.

**Table 2:** **Global interaction matrix**

	**P1**	**HC-Pro**	**P3**	**6 K1**	**CI**	**6 K2**	**VPg**	**NIaPro**	**NIb**	**CP**	**P3N-PIPO**
P1					57%		63%				
HC-Pro		78%			44%		44%	44%			
P3								56%	67%		
6 K1								44%			
CI					57%		56%	56%		50%	100%
6 K2							44%	44%			
VPg							89%	56%	56%	44%	
NIaPro								78%	78%	44%	
NIb									44%	56%	
CP										88%	
P3N-PIPO											

All interactions with a *RC* >44% are displayed in a matrix form.

#### Y2H intensity

We used the intensity data (whenever available) and tried to correlate it with the frequency of each interaction. We grouped together all the data from Y2H studies and plotted the intensity against the overall frequency of all interactions (data not shown). We found no correlation (*r* = 0.249, 45 d.f., *p-value* = 0.172) between intensity and frequency for any of the seven potyvirus studied with Y2H. This leads to the conclusion that the biological importance of an interaction (related with the frequency with which it is detected) is not function of its intensity. In other words, interactions with lower intensity can be as vital to virus development as the more intense.

#### VVPI network construction and visualization

As it was explained in the Methods section, we set a threshold of 44% in the *RC* to separate relevant interactions from the rest. With this constraint, only 26 out of the 66 possible interactions were considered as relevant. With those interactions the global interaction matrix (GLIM) was built (Table 2).

**Figure 2:**
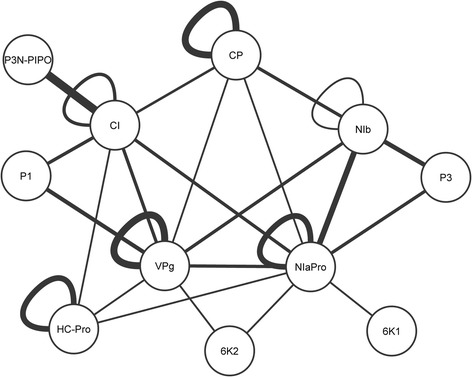
**Global interaction network.** Visual representation of the most relevant protein-protein interactions in the *Potyvirus* genus.

The network defined by GLIM shows the proteins as nodes and the interactions as edges. It represents the VVPIs detected in the studies with a *RC* >44%. Additionally, to increase the visual information the width of the edges was made proportional to the *RC* of the interactions. The resulting network (Figure 2) is the global interaction network (GLIN).

#### Interaction relevance

**Figure 3:**
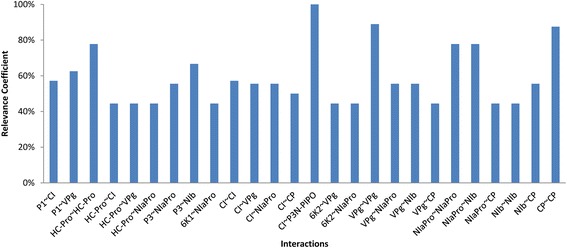
**Relevance coefficient of all interactions of the global interaction network.**

The starting point for the topological analysis is the computation of the *RC* for every interaction (with *RC* >44%) experimentally detected (Figure 3). Some interesting information arises from this representation. The most common interactions have a *RC* in the range 80% - 90% (with exception of the CI ~ P3N-PIPO). P3N-PIPO was tested only in one of the studies [44] and only against three proteins: itself, CP and CI. The positive hit of the CI ~ P3N-PIPO interaction produces a *RC* = 100% for this particular interaction. However, it is reasonable to assume that after P3N-PIPO is tested against all viral proteins in future studies, this *RC* value will decrease. Core interactions involve proteins CI, VPg, NIaPro and NIb. Out of the 66 possible interactions, 26 were considered relevant representing a striking 39.3%. This shows clearly that the intraviral network is highly connected. It is generally accepted that viral proteins are multifunctional, so this high connectivity was expected. Another interesting conclusion drawn from Figure 3 is that there is no specific *RC* threshold dividing the interactions between the most common and the rarest. In other words, there are interactions detected across all the *RC* range (from 100% to the established limit of 44%).

#### Protein connectivity

**Figure 4:**
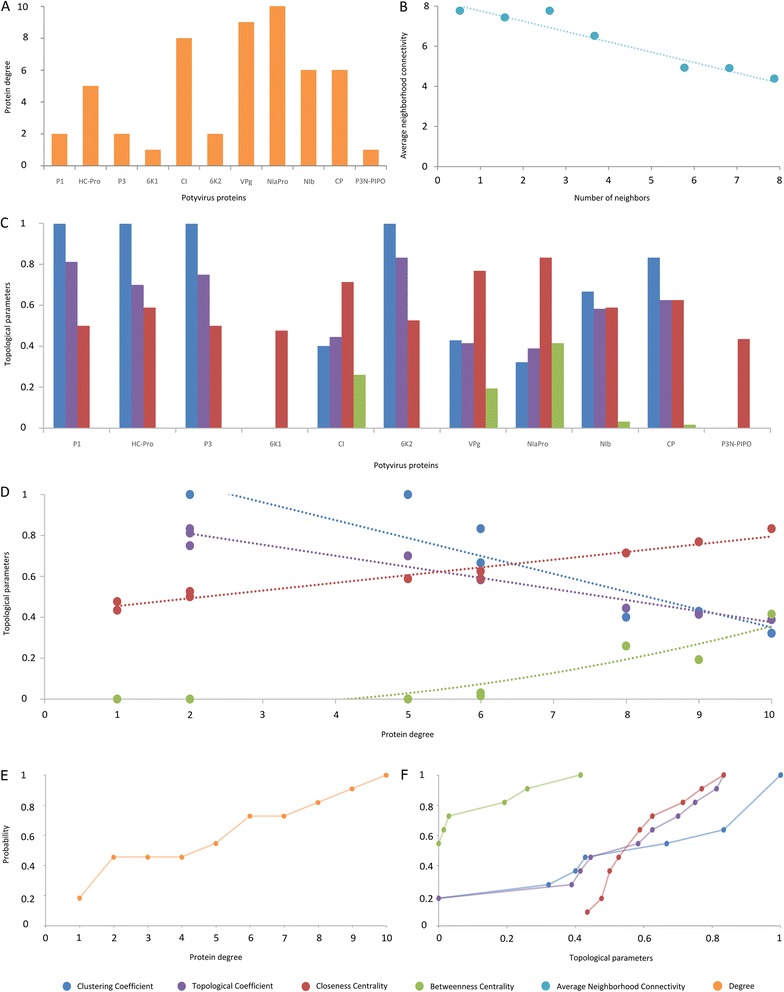
**Protein connectivity and topological analysis of the global interaction network (GLIN). (A)** Degree of each potyvirus protein. **(B)** Average neighborhood connectivity distribution. **(C)** Topological parameters of each protein. **(D)** Topological parameters of proteins related with their degree. 6 K1 and P3N-PIPO data for the clustering and topological coefficients were removed from the representation (commented in the text). **(E)** Degree cumulative probability distribution. It shows the probability that a protein has a determined degree or lower. **(F)** Cumulative probability distribution of topological parameters.

In a PPIN, the degree of each node matches the number of different interactions in which each protein is involved but only if there is no self-interaction. If there is, the protein degree equals the number of interactions plus one (see Figure 4). Supporting the idea that viral PPINs are highly connected, Figure 4A shows that the degree of most proteins is in a narrow range (2–10). However, a clear distinction can be made between high and low connected proteins. Low connected proteins are P1, P3, 6 K1, 6 K2, and P3N-PIPO, and they have a degree in the low range of 1–2. Highly connected ones are HC-Pro, CI, VPg, NIaPro, NIb, and CP, with a degree of 5–10.

Furthermore, we investigated whether there is some relation between interactions relevance and protein degree. It seemed that interactions with the highest *RC* were formed by proteins with a high degree. To check this we performed a correlation study, and we found no relation between *RC* and degree (*r* = −0.034, 24 d.f., *p-value* = 0.871). In spite of that, it is noteworthy that the five most relevant interactions (VPg ~ VPg, CP ~ CP, NIaPro ~ NIb, NIaPro ~ NIaPro and HC-Pro ~ HC-Pro) are formed by proteins with a high degree (without considering the CI ~ P3N-PIPO interaction).

It is also interesting to study the assortativity [54] of the network. Assortative mixing is the preference for the nodes of a network to attach to others that are similar. This is commonly examined in terms of a node’s degree. In PPINs, consists of studying whether high degree proteins tend to establish interactions with other high degree proteins. One way to capture the assortative behavior of a network is to examine the average neighbor connectivity. The connectivity of a node is the number of its neighbors. The neighborhood connectivity of a node is defined as the average connectivity of all its neighbors. The neighborhood connectivity distribution gives the average of the neighborhood connectivities of all nodes with *k* neighbors for *k* = 0, 1… If this function is increasing, the network is assortative, since it shows that nodes of high degree connect, on average, to nodes of high degree. On the other hand, if the function is decreasing, the network is dissortative, since nodes of high degree tend to connect to nodes of lower degree. Average neighbor connectivity distribution for the GLIN is shown in Figure 4B (Additional file 5). The values of the parameter decrease with the number of neighbors, therefore the GLIN shows a dissortative behavior. This agrees with previous studies that stated the dissortative nature of biological networks [54]. However, biological interpretation of this fact remains unclear. Hierarchical structures in biological networks may result in dissortativity. Regulatory genes or transcription factors influence many particular genes or proteins with specific biological functions. Therefore, hubs correspond to regulators and less connected nodes to actuators, dividing the network in several hierarchical levels. Among the 11 nodes in the PPIN, HC-Pro is the most highly connected component, interacting with all other nodes. Therefore, dissortativity in this network emerges as a simple consequence of the limited number of nodes and that the most connected one interacts with all other nodes, regardless their specific connectivity.

#### Topological analysis

As it was mentioned in the Methods section, a complete topological analysis of the GLIN and all its nodes was carried out. First, a set of general topological parameters was calculated for the entire GLIN (Table 3). The clustering coefficient is high and the characteristic path length is lower than two, emphasizing the fact that GLIN is highly connected. The number of self-loops is quite high (six out of 11 possible), meaning that most proteins interact with themselves for carrying out some of the biological functions.

**Table 3:** **General topological parameters of the global interaction network**

Clustering coefficient	0.605
Connected components	1
Network diameter	3
Network radius	2
Network centralization	0.533
Characteristic path length	1.745
Average number of neighbors	3.636
Number of nodes	11
Network density	0,364
Network heterogeneity	0,634
Number of self-loops	6

In addition, four topological parameters were computed for each protein in the GLIN. This topological information is displayed in Figure 4C (Additional file 6). Some parameters contain related information such as centralities and the clustering and topological coefficients. NIaPro, VPg and CI have the highest centralities and the lowest clustering and topological coefficients. A similar conclusion can be drawn from the low clustering and topological coefficients of 6 K1 and P3N-PIPO because they do not form any 3-loop in the network. P3N-PIPO is only linked to CI and 6 K1 only to NIaPro. Therefore their topological parameters are quite different from the highly connected rest of proteins (especially the clustering and topological coefficients, which are based on common neighbors). An identical analysis was performed for PPV, SMV-P and SYSV-O, since they were the only ones with enough interactions detected to construct a complete topology (see Additional file 6).

It is important to remark that these parameters are in part influenced by the degree of each protein (Figure 4A). In general, the clustering and topological coefficients increase with degree while closeness and between centrality decreases (Figure 4D). The least connected proteins have an extreme clustering coefficient (0 or 1) while the most connected ones have intermediate values. Both centralities are higher for high degree proteins, which is to be expected. HC-Pro is located somewhere in the middle. It has a high degree but its centralities are low and its topological coefficient is high. It also has an extreme clustering coefficient. Clustering and topological coefficients have the worst fitting to a linear regression due to the low degree of 6 K1 and P3N-PIPO, which was already discussed. Complete statistical description of the regressions (*p-value*, d.f. and *R*^2^) can be found in Additional file 7. It is worth noting that non-linear models have a better fit in the betweenness centrality data.

Finally, the topological distributions of the different parameters were determined, displayed and studied. Topological distributions compute the probability that a node in a network presents a particular value in some parameter. For instance, the probability of a node to have a degree of three. Although informative, they are more useful when computed as cumulative distributions. Following the example, the probability of a node to have degree lower than or equal to three. Cumulative distributions of degree and other topological parameters were calculated for the GLIN (Figure 4E and F, data in Additional file 5). The cumulative degree distribution for the GLIN shows a quasi-linear behavior. Obviously, the probability increases with the degree. The other cumulative distributions also tend to be linear.

### VHPI network analysis

In this second subsection of the Results, integration of the virus network and the host network (through VHPIs and HHPIs) was studied. VHPI network construction and visualization subsection focuses on the difficulty of the faithfully representation of networks of this size. Effect propagation deals with the effect of specific viral proteins along the HHPIN and Similarity analysis focuses on the comparing the patterns of propagation of pairs of viral proteins.

#### VHPI network construction and visualization

**Figure 5:**
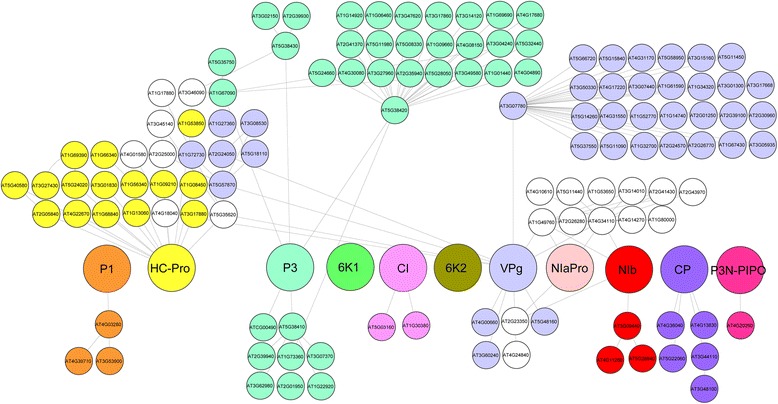
***Potyvirus*** **-** ***A. thaliana*** **VHPI network (VHPIN).** Proteins and their host neighbors are grouped by colors. White color is assigned to host proteins connected to several viral proteins during the same step. For instance, host protein At2G23350 (located just below VPg protein) is represented white because is linked directly to two different viral proteins: VPg and NIb.

Potyvirus proteins establish interactions with a large unknown number of host factors, disrupting the normal development of the plant. These VHPIs channel the harmful effect of the virus and point to the vital nodes of the PPIN and transcriptional regulatory network of the host [30]. The effect propagates from those direct VHPIs through the entire network of HHPIs. Visualization of the *A. thaliana* interactome is impossible in practical terms. It has 5127 nodes (proteins) and 12624 edges (interactions) and therefore any attempt to visually represent the network as a whole is not going to provide useful information. Instead, we chose to illustrate the 11 potyvirus proteins surrounded by two levels or steps of plant interactions [5]. This Potyvirus-*A. thaliana* VHPI network (VHPIN) (Figure 5) provides a quick overview of the anchor points that the virus uses to hijack the plant network. It is clear that the virus hits many proteins in the first step. However, the interactions vary in number and connectivity. For instance, proteins P3 and VPg hit two host proteins that are network hubs while HC-Pro directly interacts with more than 10 different proteins and then diversifies its effect to all the interactions of these proteins. The VHPIN does not show any information of the interactions happening in successive next steps (step 3, 4 and so on).

#### Effect propagation

**Figure 6:**
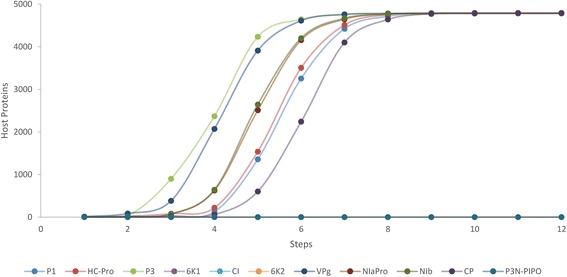
***A. thaliana*** **interactome coverage.** It shows the protein-protein interactions occurring from each potyvirus protein and going across the whole plant network.

To study the potential effect that the viral proteins have on the network, the 11 viral proteins were taken as starting point and used the *A. thaliana* interactome as a map to draw the complete tree of interactions that appear until no more interactions are possible. The first two steps are represented in the VHPIN but beyond that it is not practical to visualize the interactome as a network illustration, so we have to rely on mathematical description. For instance, the protein P1 establishes only one interaction with a plant protein (step 1), then this protein establishes two interactions with other plant proteins (step 2, the VHPIN displays the protein relationships up to this point) but the network keeps growing; these two proteins link with 13 proteins (step 3), these 13 link with 110 (step 4) and so on. We repeated these calculations for the 11 viral proteins and the results are displayed in Figure 6 and Additional file 8 (note that both the table and the figure show the cumulated number of interacting proteins). Some information may be directly extracted from the illustration. Hence, 6 K1, CI, 6 K2, and P3N-PIPO establish virtually no interactions with the host. We envision three possible explanations for this lack of interactions. (i) These proteins function only by interacting with other viral proteins but not with host factors. (ii) These proteins may interact with host proteins via other viral proteins or via other host elements such as RNA, DNA, lipids or carbohydrates. And (iii) the lack of reported interactions does not necessarily means these interactions do not exist, reflecting the need of additional work. This is the obvious case for the recently described P3N-PIPO.

The other seven proteins are able to reach essentially the whole *A. thaliana* network (around 93%). Full speed propagation starts in step two and ends around step eight. Some small sections of the network are unreachable because they are not connected to the main module. Of course, this does not mean that the effect of those seven proteins is relevant and significant in the whole plant network. The effect may loss its biological importance after a few steps of interactions unless the affected proteins are transcription factors that may function as hubs in the global regulatory network. In such case, the perturbation will be efficiently transmitted along the entire network. In all other instances, viral proteins will affect the host network only to a certain extent and possibly circumscribe their action to specific branches or modules. However, a global analysis is still useful to compare the viral proteins with one another. Some proteins such as P3 or VPg propagate their action through the network remarkably faster than others like CP or P1. This may indicate the sequential order in which the effect of the proteins crosses the network during the virus cycle. This measure of steps can be seen as a temporal variable. The effect of one viral protein is likely to be noticed earlier in a host protein located two steps away than other located six steps away. It seems reasonable to assume that, in spite of the enormous diversity and relevance of host interactions, some viral proteins act earlier than others during the infection cycle and that this kind of propagation analysis is a reasonable approach to study them.

#### Similarity analysis

Effect propagation analysis does not evaluate how similar two viral proteins are in their relationship with the host; whether they hit the same host proteins and in the same or similar number of steps. Some measure of similarity in effect propagation among viral proteins is thus needed. For example, let us assume that P1 reaches five host proteins while HC-Pro reaches 10 at a determined step, and that one of those host proteins (HP1) is common for both viral proteins. Two groups are formed: P1-group (with five members) and HC-Pro-group (with 10 members) having one member (HP1) belonging to both groups at the same time. It is possible to quantify the similarity of those two groups using a similarity coefficient such as the *SI*. It varies from 0 to 1 and expresses the similarity between two groups of proteins. We calculated it for every pair of viral proteins (55) and for all the steps (12) (Additional file 9). The *SI* was calculated as an accumulative variable. This way each value gives an idea of similar behavior up to that step. Plotting its evolution over the steps produces dynamical coinciding patterns. It tends to increase in the mid-steps because at that point the viral effects are propagating at full speed, and those interactions are usually common to most viral proteins.

**Figure 7:**
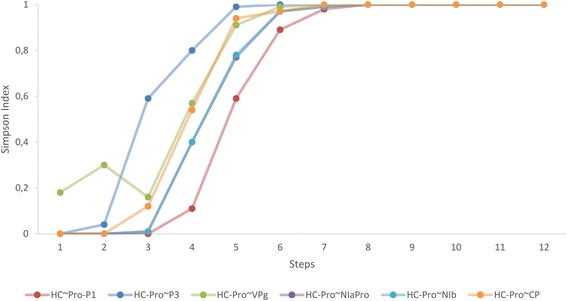
**Simpson index evolution for HC-Pro.** All possible combinations between HC-Pro and other viral proteins that propagate through the network. Differences in speed and shape of the spreading patterns for each pair can be easily observed. Straight lines link the values of the *SI* for each step representing how it varies while the protein pair effect propagates through the network.

Different features can be illustrated through *SI* graphs quite easily. We show in Figure 7 the *SI* for all proteins paired with HC-Pro. This allows us to point out interesting specific behaviors. The most common behavior for a couple of proteins is that similarity starts at zero and begins to increase around step 2–3 until it reaches its maximum at step 7–9. The first and main difference is speed; some pairs reach a high *SI* much faster (*e.g.*, HC-Pro ~ P3) than others (HC-Pro ~ P1). However, there are a few cases in which the *SI* for a pair of proteins decreases at some steps (HC-Pro ~ VPg, steps 2–3). This is somehow surprising, since the index is calculated with accumulated proteins in each step. Therefore, the networks are always increasing their size in each step. However, in some interactions (and for some steps) the networks of both proteins increase but the common host proteins to both viral proteins in that steps does not increase proportionally. Consequently there is an absolute decrease in similarity. Nonetheless, *SI* always end up increasing until a value of almost one because the seven viral proteins that propagate their effect all reach the entire host network.

**Figure 8:**
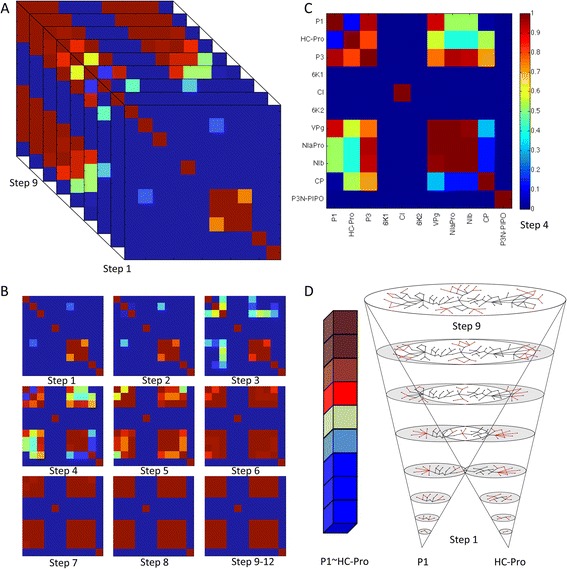
**Voxel representation of the Simpson index. (A)** Voxel representation of the Simpson index for the viral proteins across the HHPIN. **(B)** Consecutive pixel representations of the *SI* for the twelve steps that form the HHPIN. **(C)** Pixel representation for step 4. Viral proteins are shown in X and Y axes and relevance coefficient color legend is displayed on the right side vertical axis. **(D)** Evolution of the *SI* for the P1 ~ HC-Pro interaction across the entire HHPIN. A schematic cone of possible interactions is displayed as well to visually represent the networks growing from the viral proteins (step 1) until the end of the HHPIN.

The information drawn from this similarity analysis complements the effect propagation study shown before. However, even for pairs of proteins, representing visually similarity is not trivial. Similarity evolution for a specific pair of proteins can be easily plotted but displaying all of them at the same time, while retrieving useful biological information, is much more difficult. To tackle this we used voxel-based representations. We constructed a 3-dimensional matrix called voxel to visually represent the evolution of the *SI* over the host-host protein interaction network (HHPIN). The first two dimensions represent the eleven viral proteins; this creates a grid that assigns a pixel to each pair of viral proteins. The main diagonal has no biological meaning because the similarity of a protein with itself is always one. Furthermore, the information is repeated twice in the grid (P1 ~ HC-Pro pixel contains obviously the same information as the HC-Pro ~ P1 pixel). The color of the pixel represents the value of the *SI* for that particular combination. The third dimension is the distance (measured in steps) from the original viral pair of proteins to any particular point in the HHPIN. This representation (Figure 8) allows any viewer to find quickly the spaces of interest: which viral proteins link with the host, in which steps the *SI* changes the most, which pairs of proteins follow a determined evolution, etc. Additionally the projection of each pixel over the steps (Figure 8D) reveals the particular evolution of the *SI* for that pair of proteins.

## Conclusions

Topological properties of the potyvirus PPIN were studied in great detail. Data was collected from different sources and was processed and integrated in the intraviral network representation GLIN. Our findings confirm the idea that intraviral network of potyvirus is highly connected and core interactions involve proteins NIaPro, VPg, CI, CP, and NIb. The four topological parameters studies seem to depend on the protein degree. Moreover, the cumulative distributions of these parameters and the degree increase in a quasi-linear way. BiFC and Y2H offer similar results and detect the most common interactions. Y2H data led us to affirm that interactions with lower intensity can be as vital to virus development as the more intense ones.

In the study of host-virus interaction, VHPIN results an accurate representation of the plant-host interactome. Proteins P3 and VPg focus their effect in only one hub while HC-Pro diversifies its effect among several proteins through direct interactions. Viral proteins differ in the efficiency in which their perturbations are transmitted throughout *A. thaliana* HHPIN. Proteins P3 and VPg are the fastest to propagate their effects while proteins CP and P1 are the slowest ones. The similarity among viral proteins in their patterns of perturbation transmission was analyzed using the evolution of the Simpson index (*SI*) along propagation steps. This analysis highlighted common patterns of action between NIaPro, NIb, VPg, and P3.

This study opens new research avenues. This topology can be used as a base for a much more in-depth analysis of virus development with the addition of biological meaningful measures such as virus growth or fitness. On the other hand, the VHPIN analysis can be further explored using more complex metrics, graph kernels or integrating more biological information available such as sub-cellular localization or biological function. Additionally, when more studies start to use the BiFC method and the pool of reliable intravirus interactions tested and detected increases, the topology here determined can be slightly modified to meet the new data.

## Additional files

Additional file 1:
**Initial potyvirus proteins interaction dataset (VVPIs).** Dataset of potyvirus proteins interactions gathered from six different sources. Additional file 2:
***A. thaliana*** **interactome (HHPIs).**
Additional file 3:
**Dataset of potyvirus-** ***A. thaliana*** **interactions (VHPIs).**
Additional file 4:
**Common pool of potyvirus protein-protein interactions (VVPIN).** The first sheet shows the complete original dataset grouped in the different interactions. The second sheet presents the dataset once it has been symmetrized as it is explained in the main text. Additional file 5:
**Cumulative topological distributions for the global interaction network (GLIN).** It contains the distributions for degree, clustering coefficient, topological coefficient, closeness centrality, betweenness centrality and average neighborhood connectivity. Additional file 6:
**Topological parameters of the global interaction network (GLIN) and other three viruses networks.** It contains general parameters for the entire networks and for each particular protein in each network. Additional file 7:
**Statistical description of the regressions and correlations performed in the study.**
Additional file 8:
***A. thaliana*** **interactome coverage.** Starting from each viral protein the table shows how many interactions they reach in each step. Additional file 9:
**Simpson index evolution for each pair of viral proteins.**

## Abbreviations

AP-MS: Affinity purification coupled with mass spectrometry
BiFC: Bimolecular fluorescence complementation
GLIM: Global interaction matrix
GLIN: Global interaction network
HHPI: Host-host protein interaction
HHPIN: Host-host protein interaction network
PPI: Protein-protein interaction
PPIN: Protein-protein interaction network
*RC*: Relevance coefficient
*SI*: Simpson index of similarity
VVPI: Virus-virus protein interaction
VHPI: Virus-host protein interaction
VHPIN: Virus-host protein interaction network
Y2H: Yeast two-hybrid
